# KNEE OSTEOARTHRITIS: PERCEPTIONS OF PSYCHOSOCIAL CONSEQUENCES, PAIN, AND MANAGEMENT COMPLEXITIES

**DOI:** 10.1093/geroni/igae098.3016

**Published:** 2024-12-31

**Authors:** Mark Luborsky, Marc Hochberg, Alice Ryan, Yu Dong, Brock Beamer, Michelle Shardell, Alan Rathbun

**Affiliations:** Wayne State University, Detroit, Michigan, United States; University of Maryland School of Medicine, Baltimore, Maryland, United States; University of Maryland School of Medicine, Baltimore, Maryland, United States; University of Maryland School of Medicine, Baltimore, Maryland, United States; University of Maryland School of Medicine, Baltimore, Maryland, United States; University of Maryland School of Medicine, Baltimore, Maryland, United States; University of Maryland School of Medicine, Baltimore, Maryland, United States

## Abstract

Knee Osteoarthritis (KOA) afflicts 27 million U.S. adults and has adverse health and psychosocial outcomes. Depression is twice as common among persons with KOA compared to the general population and is a barrier to pain management and physical activity. While guidelines advise depression treatment, directions on co-managing depression and pain are seldom offered; a viable adjunctive treatment, duloxetine, treats both depression and pain, yet is not routinely prescribed and could facilitate physical activity. Progress in reducing KOA distress is hampered by inadequate knowledge of perceptions and experiences of persons’ daily living with KOA especially when pain and depression co-occur. Qualitative interviews were conducted at baseline in a program evaluating aerobic exercise plus duloxetine to learn participants’ perceptions, challenges, and how depression and pain interact in managing KOA. The sample included: n=12 (5 African American, 1 Asian, 6 White) participants, aged 41-71 years (10 female, 2 male) with self-report pain severity, median 3 (scale 0-4) and moderate to severe difficulty with Activities of Daily Living. Analytic strategies featured topic, theme, and critical case comparative methods. Results identified four key constructs: (a) unpredictability and temporal variability of impacts of KOA; (b) management requiring flexible strategies encompassing a wide range of self-image and life domains beyond pain and depression; (c) barriers including concerns with medications; depression eroding energy; a sense of isolation socially and from meaningful activities; and (d) challenges to program participation. Pathways to address the constellation of intersecting domains perceived in living with KOA and treatment program participation are needed.

